# Typewriter tinnitus with time-locked vestibular paroxysmia in a patient with cerebellopontine angle meningioma

**DOI:** 10.1007/s00415-023-11869-x

**Published:** 2023-07-21

**Authors:** Yuzhong Zhang, Marcus L. F. Janssen, Erik D. Gommer, Qing Zhang, Raymond van de Berg

**Affiliations:** 1 Department of Otorhinolaryngology and Head & Neck Surgery, Maastricht University Medical Center, Maastricht, The Netherlands; 2 Department of Otorhinolaryngology, Head and Neck Surgery, Second Affiliated Hospital of Xi’an Jiaotong University, Xi’an, China; 3 Department of Otorhinolaryngology, Head and Neck Surgery, Xinhua Hospital, Shanghai Jiaotong University School of Medicine, Shanghai, China; 4 Department of Clinical Neurophysiology, Maastricht University Medical Center, Maastricht, The Netherlands; 5 School for Mental Health and Neuroscience, Maastricht University, Maastricht, The Netherlands

Dear Sirs,

Meningioma is the second most common tumor in the cerebellopontine angle (CPA), accounting for about 10% of these tumors [1]. Patients with a CPA meningioma frequently experience symptoms, such as dizziness, vertigo, unsteadiness, hearing loss, and tinnitus [1]. However, only a few patients demonstrate symptoms of typewriter tinnitus with time-locked vestibular paroxysmia [2]. Previously, a case report was described in which typewriter tinnitus was associated with vestibular paroxysmia [3]. In addition, our case report presents for the first-time video-oculography recordings and EEG findings of a patient with typewriter tinnitus and associated vestibular paroxysmia, resulting from a CPA meningioma.

A 65-year-old man presented to a tertiary referral center for vestibular disorders. He reported spontaneous short attacks of tinnitus in his right ear, with co-occurrence of unsteadiness. Symptoms progressed after radiotherapy of a meningioma (Fig. 1a) in his right CPA (maximum diameter of 19 mm), more than 1 year ago. The patient described the tinnitus as a crackling sound. These combined attacks of crackling tinnitus and unsteadiness attacks lasted less than a minute and occurred more than a hundred times each day. The attacks differed in intensity: in case of an attack with severe tinnitus, the unsteadiness also increased.

**Fig. 1:**
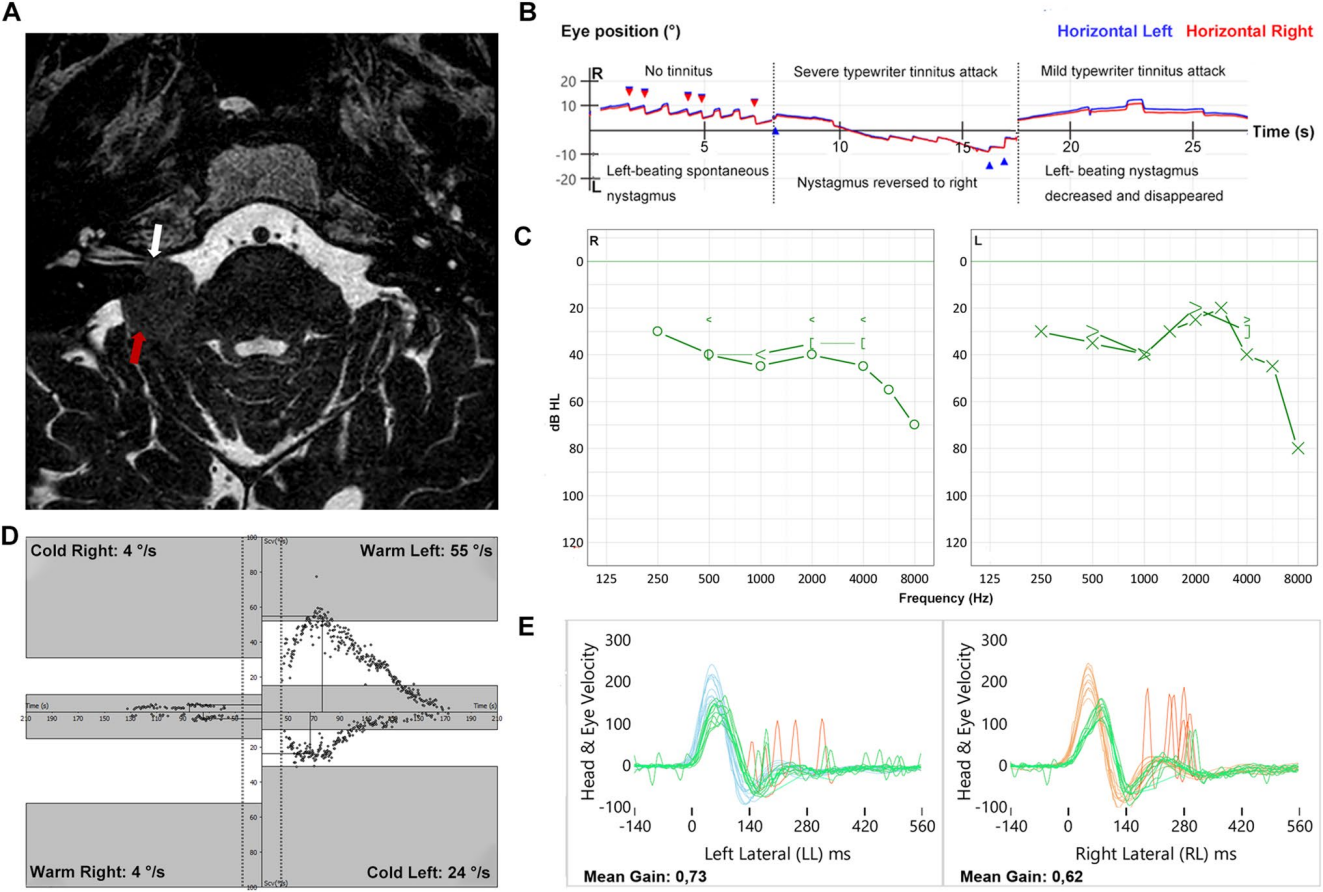
Test findings in a patient with attacks of typewriter tinnitus and time-locked secondary vestibular paroxysmia. **a** MRI demonstrating the conflict between the vestibulocochlear nerve (white arrow) and meningioma (red arrow) in the right CPA. **b** Video-oculography nystagmus recordings during (severe and less severe) attacks, and when no attacks were present. **c** Audiometric results. **d** Caloric test results. **e** Video-head impulse test results

Physical examination including Video Frenzel Goggles (VisualEyes, Interacoustics, Denmark) revealed a spontaneous horizontal left-beating nystagmus. However, during severe tinnitus attacks, reversal of nystagmus occurred into a horizontal right-beating nystagmus. This nystagmus was time-locked to the tinnitus and became left-beating again after the attack (Fig. 1b and Supplementary Video). In case of less severe attacks, the spontaneous horizontal left-beating nystagmus disappeared, or its velocity decreased (Fig. 1b). Other neuro-otological physical examinations were without any significant abnormalities, except a positive head impulse test to the right.

Pure-tone audiometry indicated a sensorineural hearing loss with a Modified Fletcher Index of 43 dB HL in the right ear and 35 dB HL in the left ear (Fig. 1c), and a maximum speech recognition score of 84% at 80 dB on the right and 100% at 70 dB on the left. The bithermal caloric test (Atmos Medizin Technik GmbH, Germany) revealed a canal paresis of 81% on the right side (Fig. 1d). The video head impulse test (Otometrics, Denmark) of the horizontal semicircular canals showed a decreased vestibulo-ocular reflex gain with covert saccades on both sides (0.62 on the right, 0.73 on the left) (Fig. 1e). MRI illustrated a conflict between the vestibulocochlear nerve and the CPA meningioma (Fig. 1a).

The following diagnosis was made: typewriter tinnitus with time-locked secondary vestibular paroxysmia, most likely due to a conflict between the vestibulocochlear nerve and the CPA meningioma. The patient was treated with 400 mg of carbamazepine per day. After 1 month, the patient indicated that the tinnitus improved significantly, and the unsteadiness completely disappeared.

Additionally, EEG recordings were performed. While the tinnitus sound was probably generated at the VIII cranial nerve, the patient only perceived the sound once the auditory sensations reached the cerebral cortex. Here, we aimed to provide an objective measure for hearing the tinnitus sound. EEG was recorded during two sessions. The initial recording was shortly after presentation before start of treatment. The patient was asked to extend his right index finger when an attack occurred, and, in addition, his left index finger in case of a severe attack. The second recording was performed 80 days later after the patient was adequately treated with carbamazepine. The patient was again asked to extend his left finger when a tinnitus episode occurred. To rule out that the EEG changes were caused by the finger movement, an additional control experiment was conducted during the second recording. The patient was also asked to extend his right index finger (71 times) upon command (while no attack was present).

EEG was acquired using a cap with 21 electrodes placed according to the international 10–20 system with Braintronics amplifiers and BrainRT software (OSG, Belgium, v4.03.00). Data were processed in Matlab (Mathworks, version2022b) using the EEGLAB toolbox (v2021.0). After band-pass filtering between 0.5 and 50 Hz, data were re-referenced to a common average reference. Electrode F8 was excluded from the first session because of artefacts. An independent component analysis artefact rejection procedure was performed to remove artefacts from eye blinks and muscle activity. Data were then segmented and aligned according to the finger extension by the subject indicating the onset of tinnitus or upon command. Data epochs were extracted from -10 s until + 30 s relative to the onset. Baseline removal was done based on the first second of each epoch. After visual inspection, epochs with artefacts were removed. Time–frequency analysis was performed across epochs using Wavelet-based analysis.

During the initial recording before treatment, beta and low gamma activity increased after the onset of the tinnitus in the parieto-temporal regions (Fig. 2a). Interestingly, after treatment with carbamazepine (Fig. 2b) and in the control experiment (Fig. 2c), a decrease in beta and low gamma was observed in the parieto-temporal regions. The increase of beta and low gamma activity was mainly present in the parieto-temporal regions where hearing is processed. It was ruled out that this was caused by the finger movement, as evidenced by the control condition (see Supplementary Figures for results of the time–frequency analysis for all EEG channels).

**Fig. 2:**
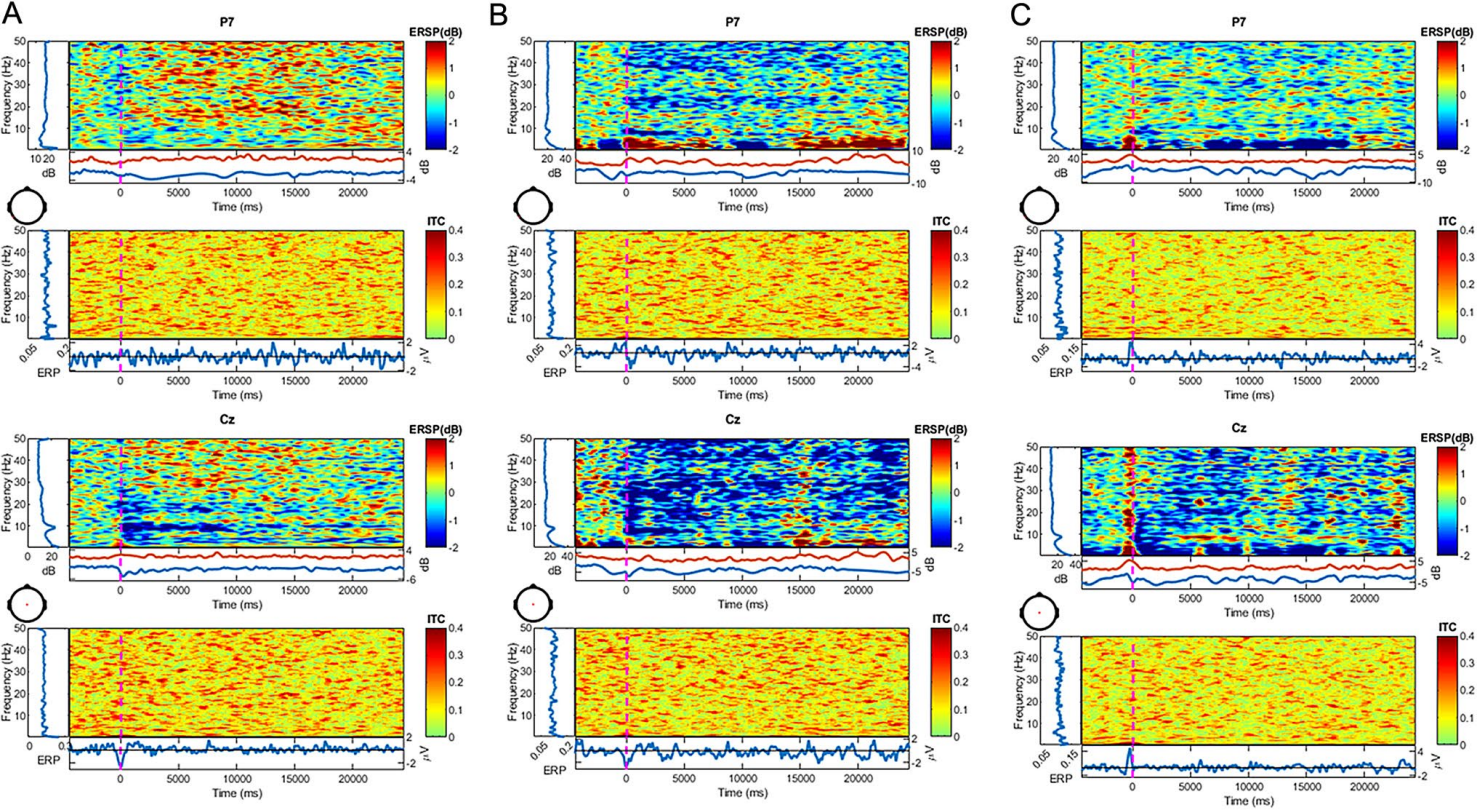
EEG time–frequency results for tinnitus onset and control. Results for the time–frequency analysis of the EEG averaged time-locked to the onset of tinnitus (*t* = 0 s) for channels P7 and Cz, respectively, for the initial recording (**a**) recording after treatment (**b**) and control (**c**) are shown (for all channels see Supplementary Figures). Per channel/component, each upper plot shows the event-related power spectrum (ESRP) and each lower plot shows the inter trial coherence (ITC). The red line below the upper plot shows the max power values in dB and the blue line shows the min power values in dB. The blue line below the lower plot shows the event-related potential in µV (ERP). Please note the increase in the power of beta and gamma band activity during the attack (**a**), this effect was reversed after treatment with carbamazepine (**b**) and neither present during a control task (**c**)

This case report presents for the first-time video-oculography nystagmus combined with EEG recordings in a patient with attacks of typewriter tinnitus with time-locked secondary vestibular paroxysmia. A horizontal nystagmus was found which changed direction over time. Most likely, these findings can be explained by vector summation of (1) a spontaneous horizontal left-beating nystagmus due to an uncompensated vestibular loss on the right side (as a result of the meningioma), and; (2) an irritative horizontal right-beating nystagmus related to the vestibular paroxysmia. During severe attacks, the irritative right-beating nystagmus was able to ‘overrule’ and counteract the spontaneous left-beating nystagmus, leading to a right-beating nystagmus. In less severe attacks, the irritative right-beating nystagmus was only able to suppress the spontaneous left-beating nystagmus, leading to a temporary absence of nystagmus. This ‘vector summation’ of different types of nystagmus was previously described in patients with a vestibular implant [4]. Although periodic direction changing of nystagmus can also be related to ‘periodic alternating nystagmus’, this diagnosis was not made. After all, the pattern of nystagmus reversal in this patient was irregular, and it was clearly accompanied by crackling tinnitus, suggesting an origin related to the vestibulocochlear nerve, and not the cerebellum [5].

To our knowledge, the EEG findings show for the first time an objective measure that resembles the perceived tinnitus sound at the cortical level. One thus could consider the paroxysmal typewriter tinnitus as the presentation of a sound. A reduction of temporal alpha power is normally seen as response to sound presentation [6]. Interestingly, only an increase in the beta and low gamma bands was observed before treatment, while after treatment with carbamazepine and in the control condition, a suppression of these bands was found. No effects in the alpha band were observed. Taking these results into account, in the future, EEG might be used as an objective marker for patients suffering from paroxysmal hearing or vestibular disorders. One should not easily extrapolate the EEG findings from this case of typewriter tinnitus to patients suffering from chronic tinnitus, since this form of paroxysmal tinnitus does not share a similar underlying pathophysiological mechanism as chronic tinnitus. After all, EEG findings in chronic tinnitus do not show a similar response: electrophysiology studies of patients with tinnitus show opposing results [6, 7].

In this case report, the attacks of unsteadiness were classified as ‘secondary vestibular paroxysmia’. After all, vestibular paroxysmia is primarily diagnosed based on typical findings and ruling out other etiologies that might mimic vestibular paroxysmia [8]. Since a CPA meningioma compressing or stretching the vestibulocochlear nerve can cause symptoms similar to vestibular paroxysmia [2], it was decided to use the term ‘secondary vestibular paroxysmia’. This implies that symptoms of vestibular paroxysmia are related to an underlying disorder, similar to, e.g., bilateral vestibulopathy resulting from meningitis [9]. Secondary vestibular paroxysmia might especially be considered in cases with abnormal test findings like spontaneous nystagmus, abnormal head impulse test, and abnormal audiometric results, because these findings are infrequent in primary vestibular paroxysmia [2, 8, 10]. Furthermore, in this patient, the typewriter tinnitus shared most likely the same etiology as the secondary vestibular paroxysmia. Additionally, demyelination resulting from radiation therapy could also be a contributing factor [11, 12].

This patient responded very well to carbamazepine treatment. Carbamazepine and oxcarbazepine are markedly effective in treating typewriter tinnitus and vestibular paroxysmia [3]. Unfortunately, around 60% of patients might stop treatment because of the side effects [13]. It is hypothesized that the mechanism of carbamazepine treatment of typewriter tinnitus is related to the inhibition of voltage-gated sodium channels, thereby decreasing the hyperexcitability of the vestibulocochlear nerve [14, 15].

Our case illustrates that CPA tumors can cause typewriter tinnitus and time-locked secondary vestibular paroxysmia. Patients with secondary vestibular paroxysmia benefit from carbamazepine therapy. In the future, EEG could be used as an objective marker for patients with paroxysmal hearing and/or vestibular disorders.

### Supplementary Information

Below is the link to the electronic supplementary material.

Supplementary file1 (DOCX 5575 KB)

Supplementary file2 (MP4 59733 KB)

## Data Availability

The authors confirm that the data supporting the findings of this study are available within the article and its supplementary materials.
