# Supplementary material for: Typewriter tinnitus with time-locked vestibular paroxysmia in a patient with cerebellopontine angle meningioma
Source: J Neurol. 2023 Jul 21;270(11):5645–8. doi: 10.1007/s00415-023-11869-x (PMC10576677; doi:10.1007/s00415-023-11869-x)
Supplement: Supplementary file 1 — Supplementary file1 (DOCX 5575 KB) [file 415_2023_11869_MOESM1_ESM.docx]

**Supplementary Figures**

Full results for the time-frequency analysis of the EEG averaged time-locked to the onset of tinnitus (t=0s) for all channels are presented for initial recording (**Supplementary Fig. 1**) recording after treatment (**Supplementary Fig. 2**) and control (**Supplementary Fig. 3**). Per channel/component each upper plot shows the event related power spectrum (ESRP) and each lower plot shows the inter trial coherence (ITC). The red line below the upper plot shows the max power values in dB and the blue line shows the min power values in dB. The blue line below the lower plot shows the event related potential in µV (ERP).

Please note the increase in the power of beta and gamma band activity during the attack, which is most prominent in the temporo-parietal regions (**Supplementary Fig. 1**). This effect was reversed after treatment with carbamazepine and a power reduction in the beta and gamma band was observed (**Supplementary Fig. 2**). The control experiment also showed a decrease in beta and gamma power. This illustrates that the increase in gamma and beta activity during the initial recording during the tinnitus attacks was indeed related to tinnitus and not related to the finger movement (**Supplementary Fig. 3**).


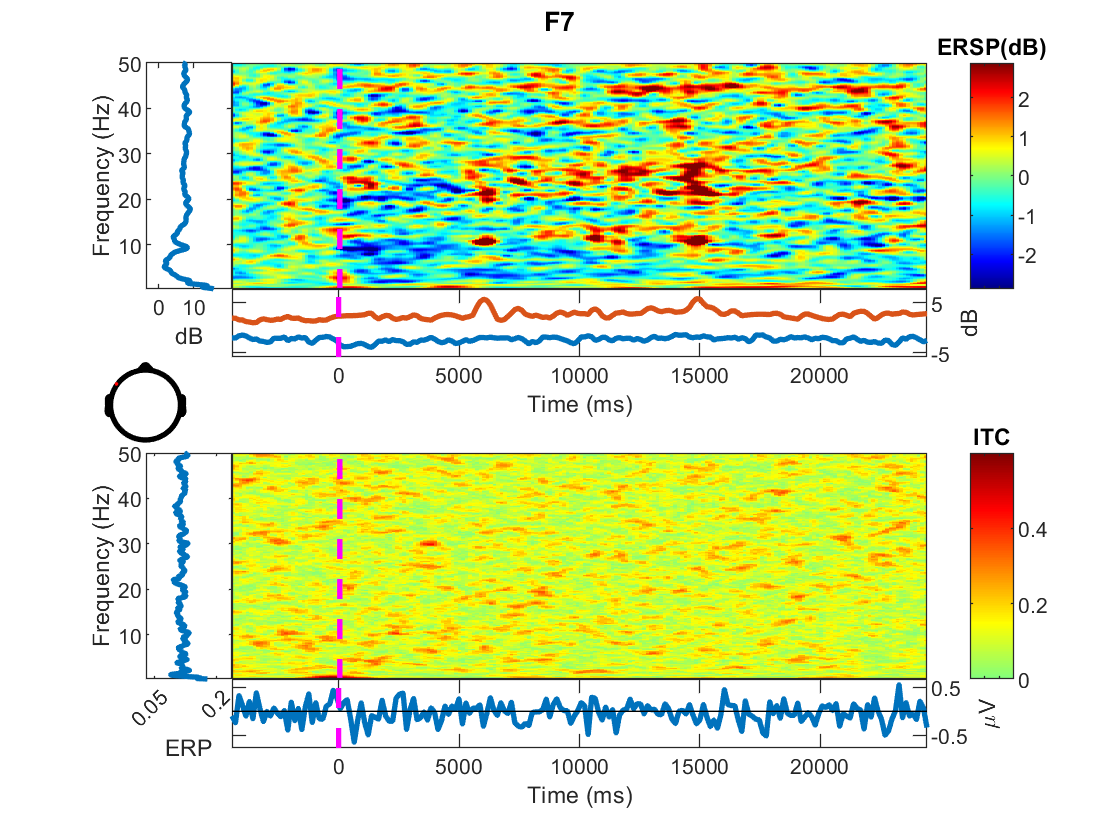

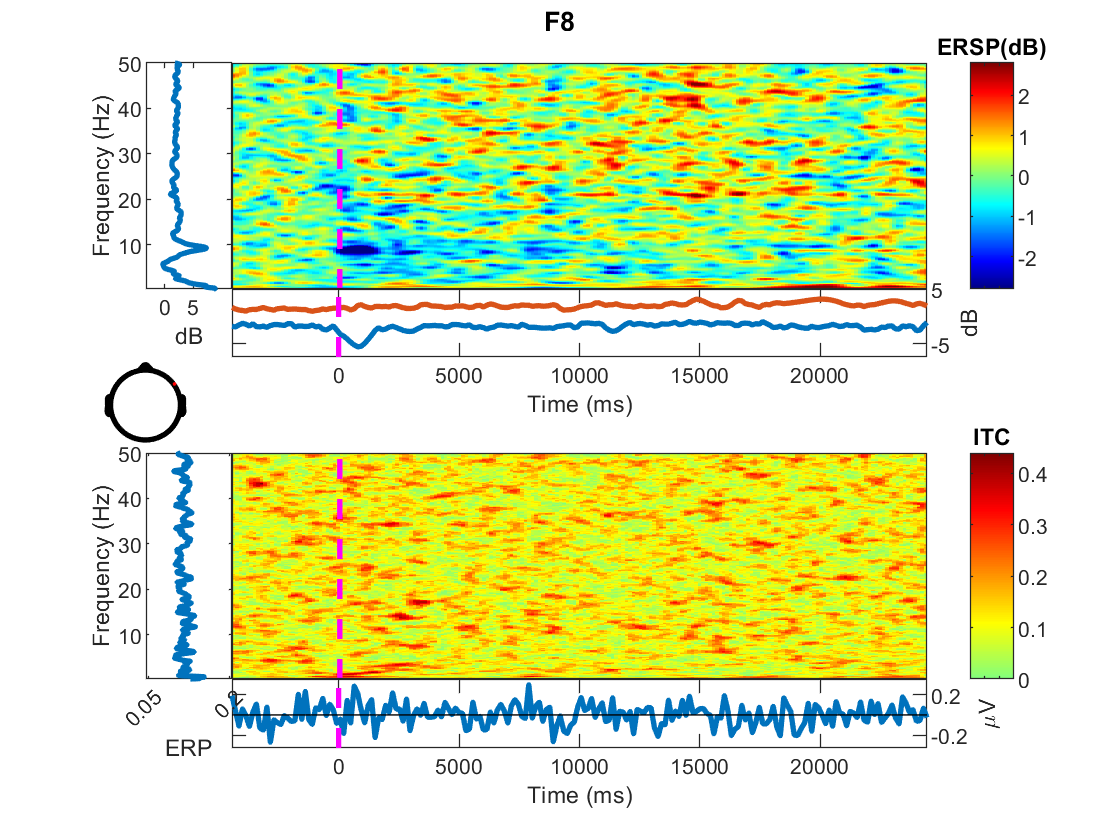

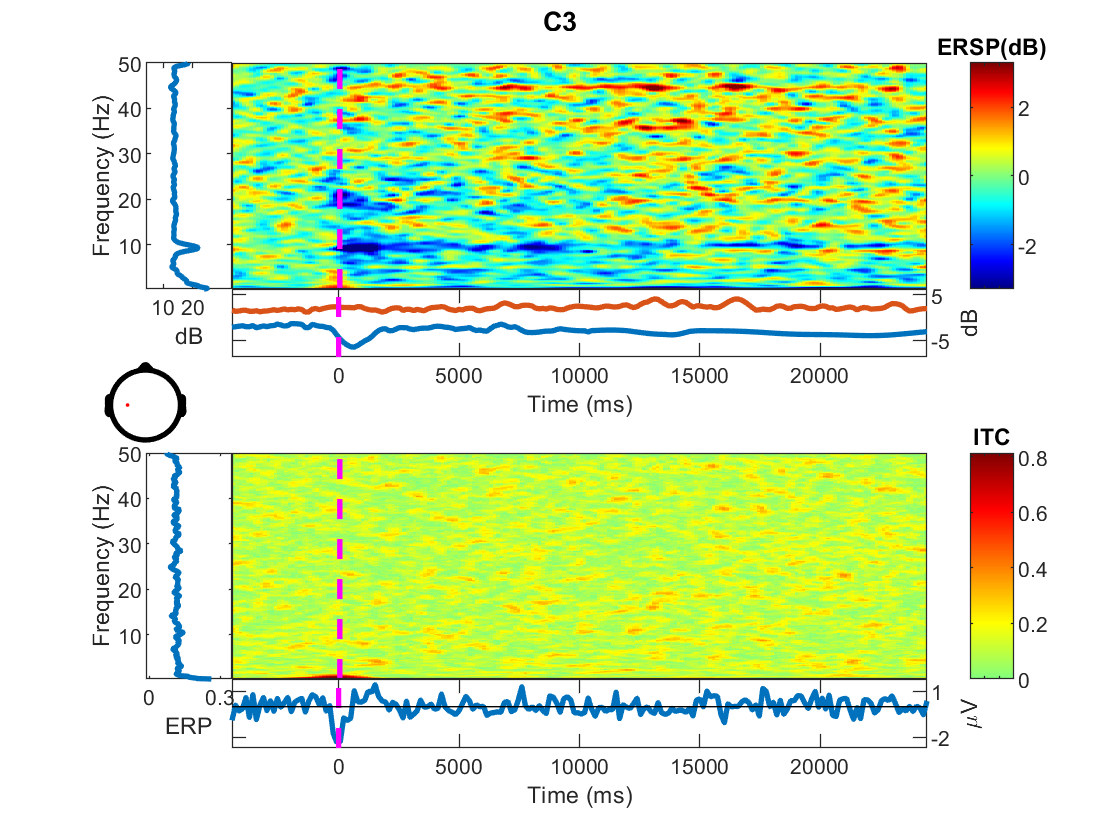

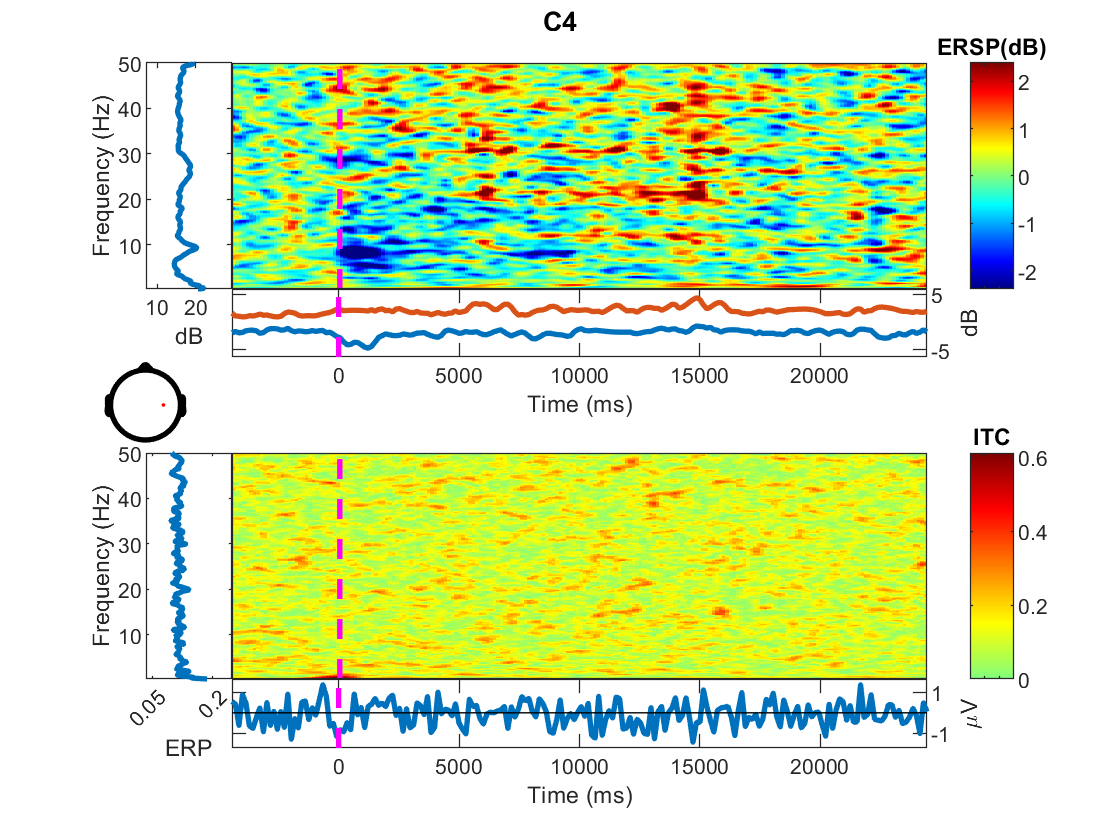

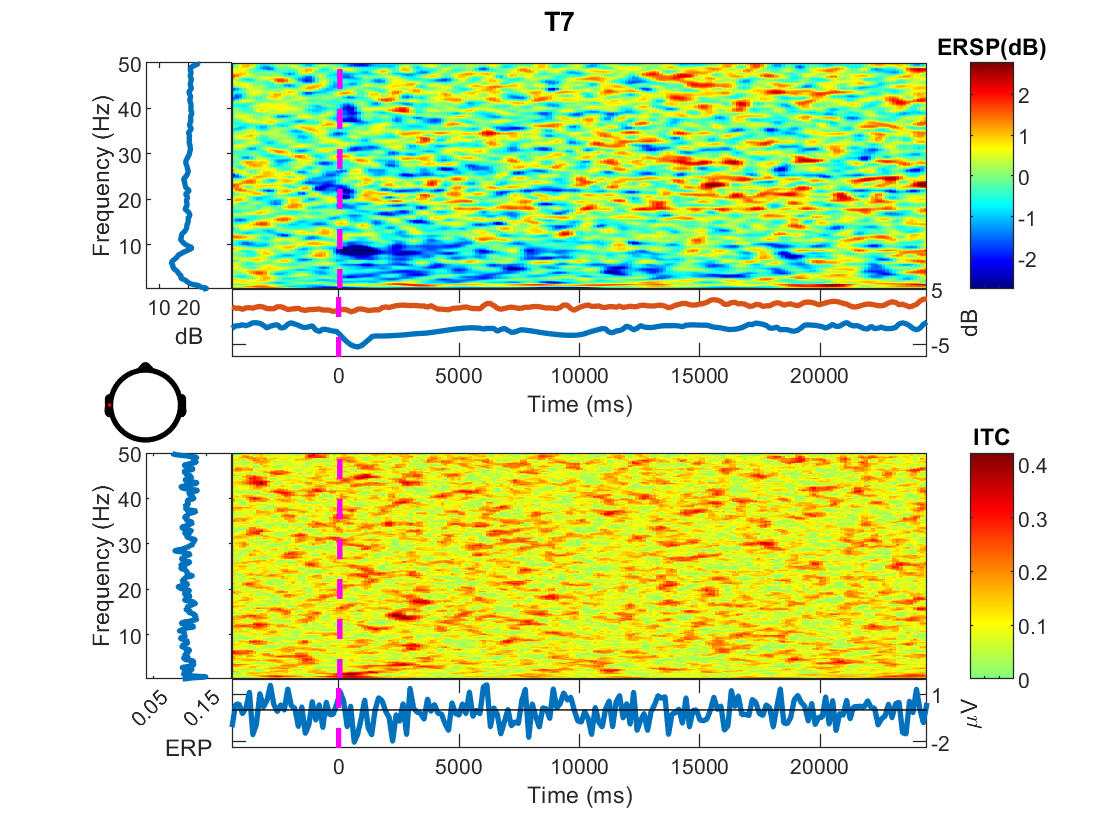

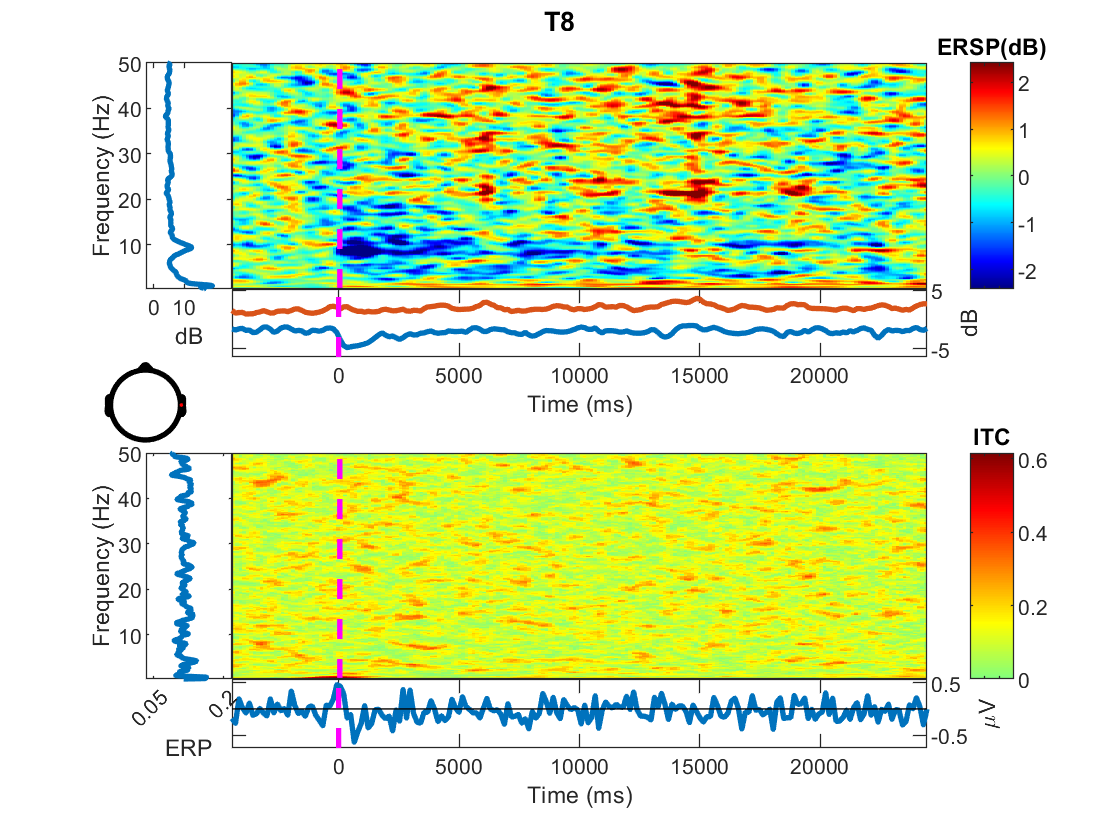

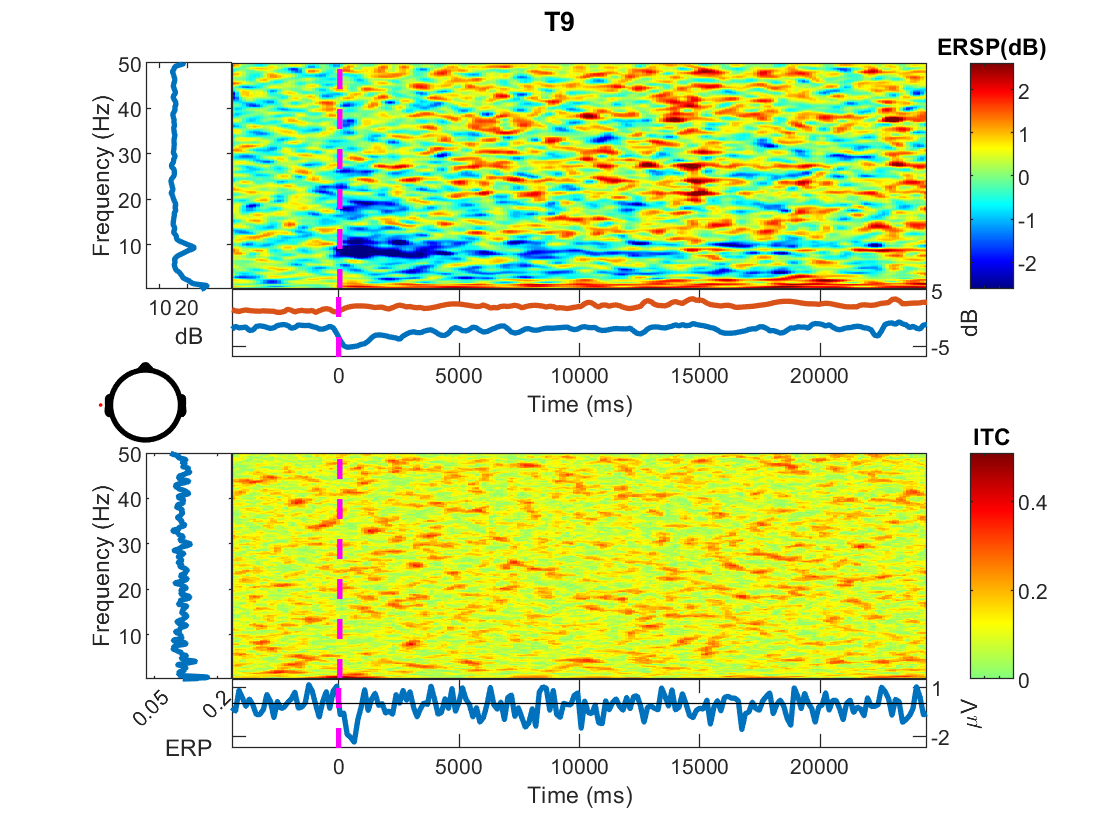

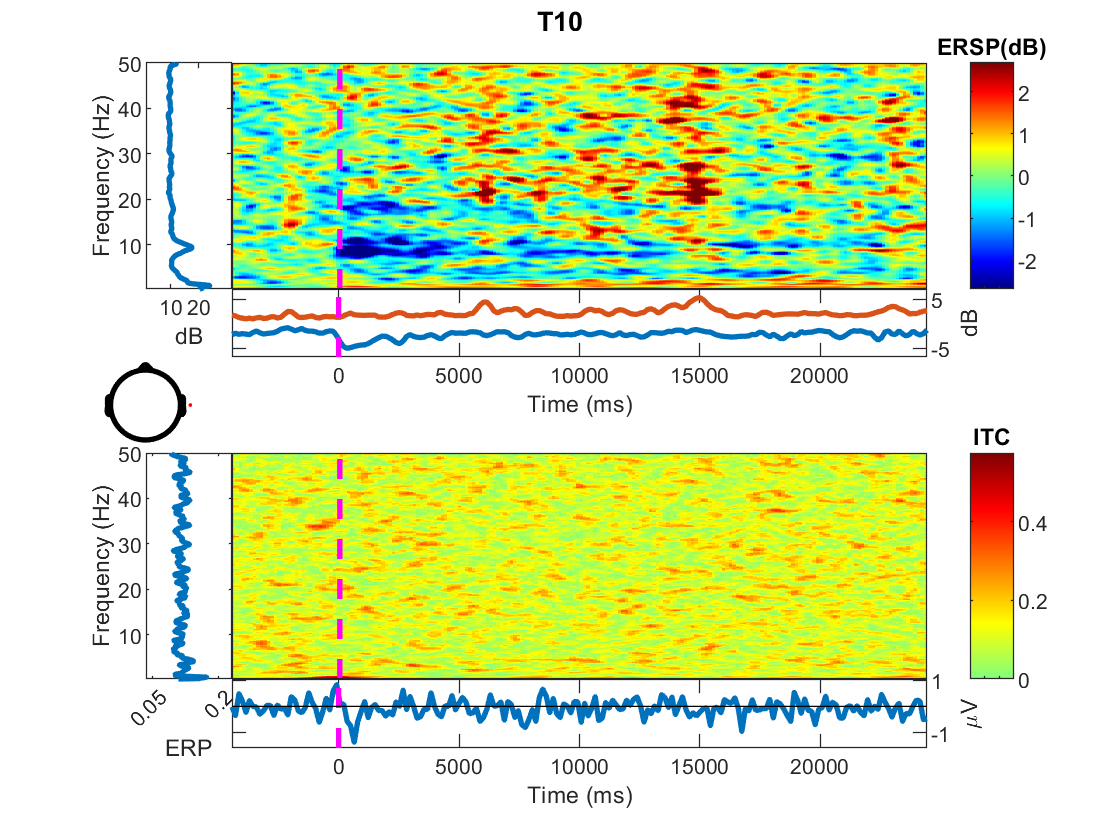

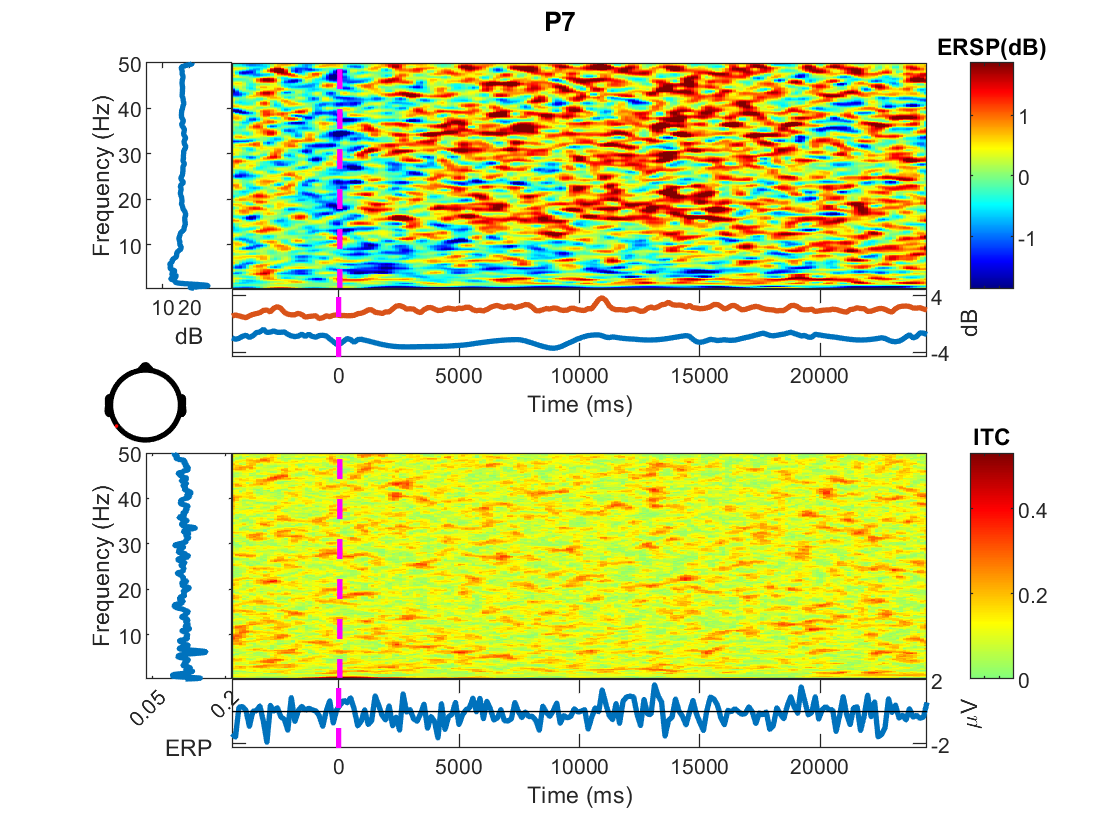

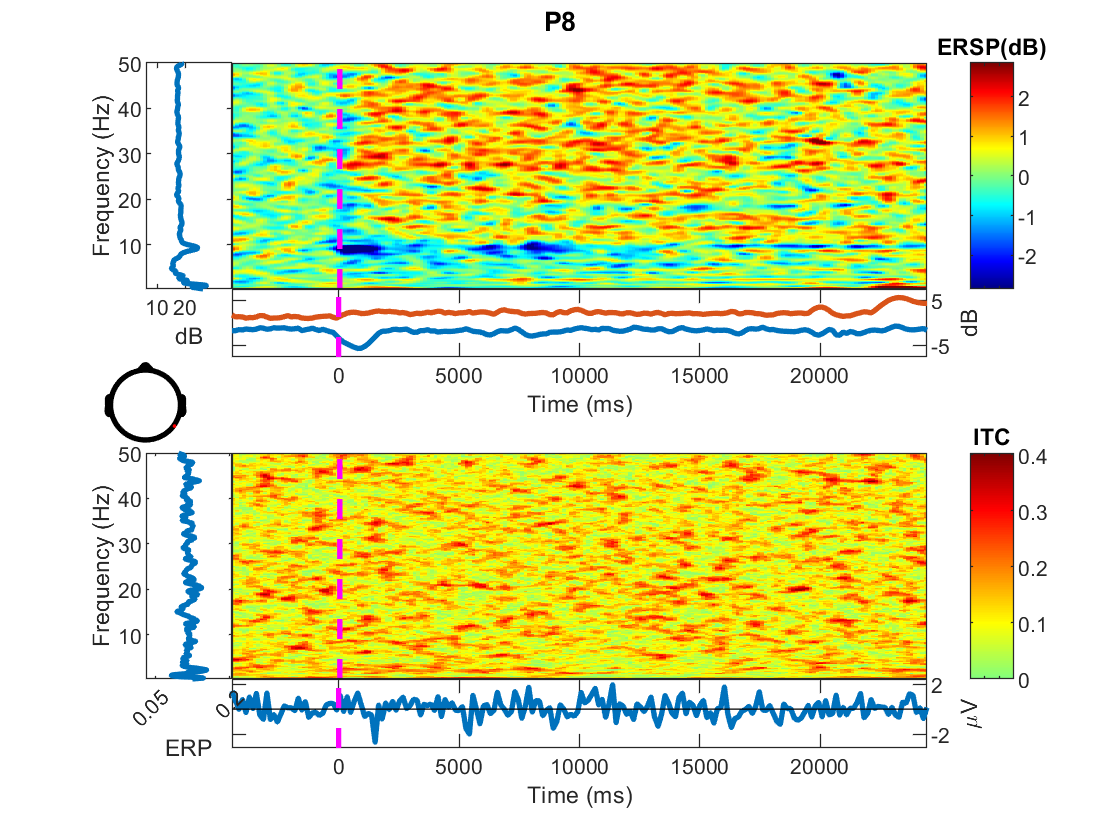

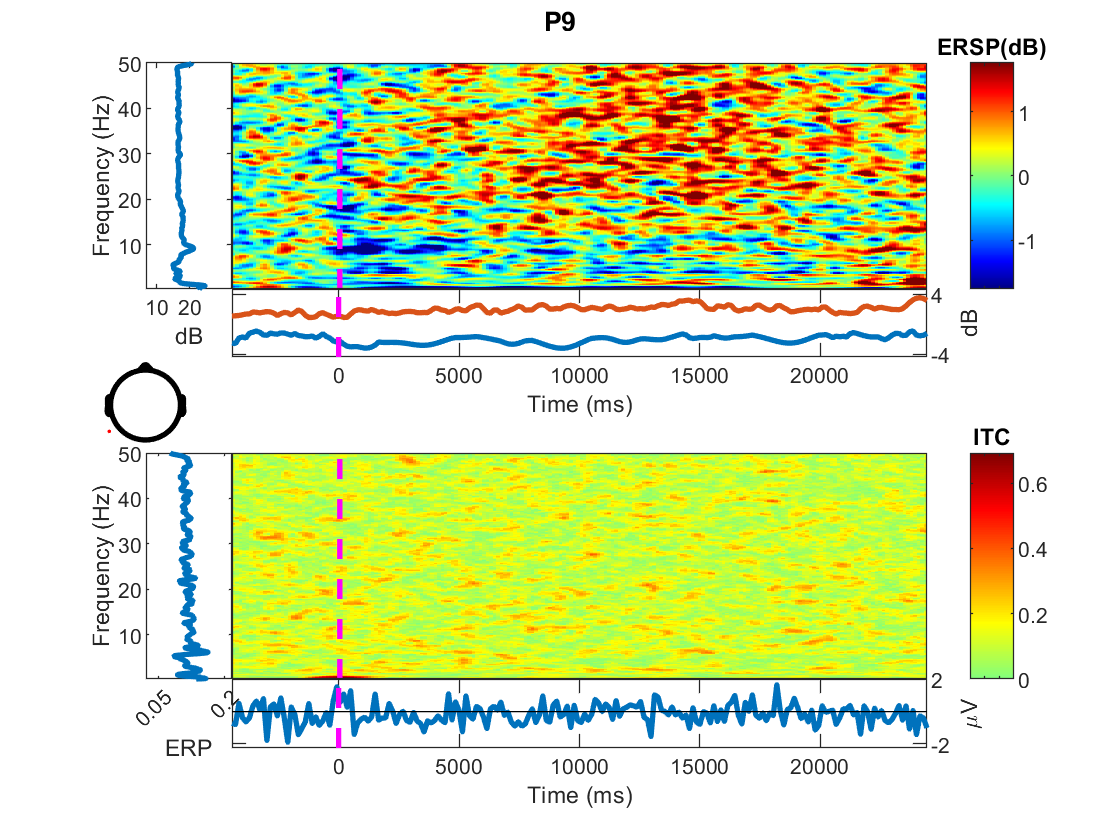

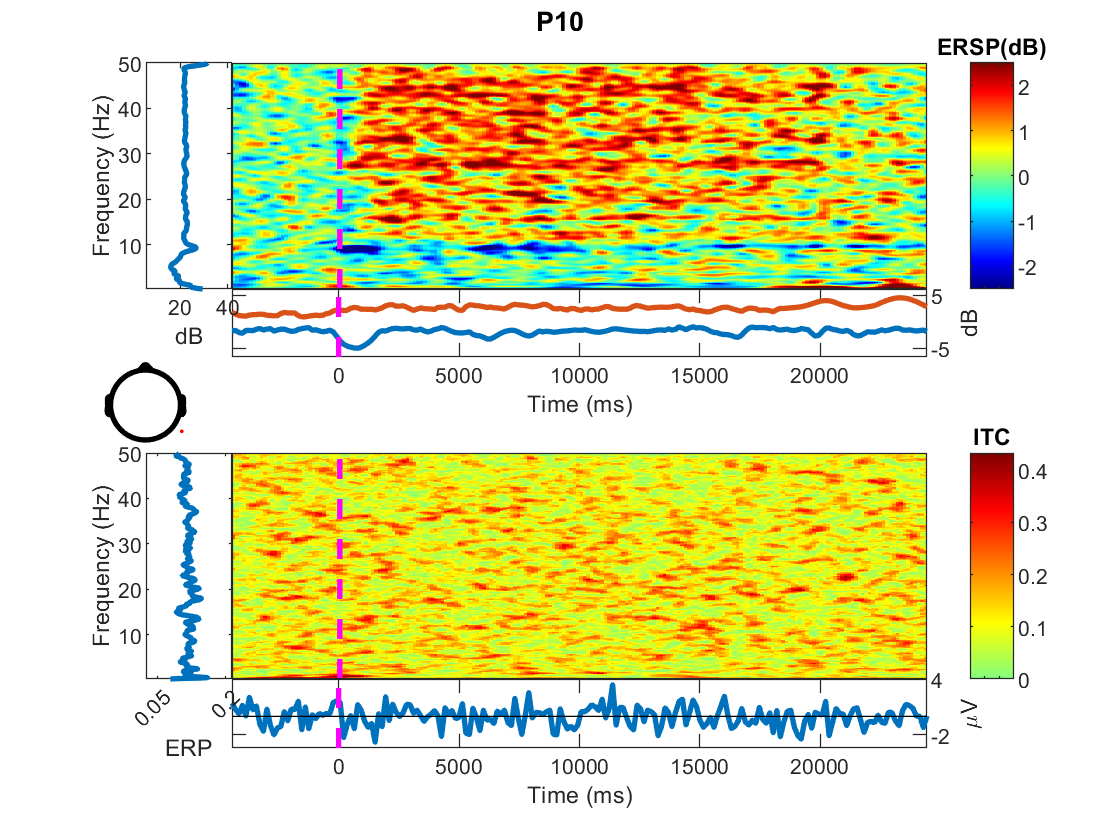

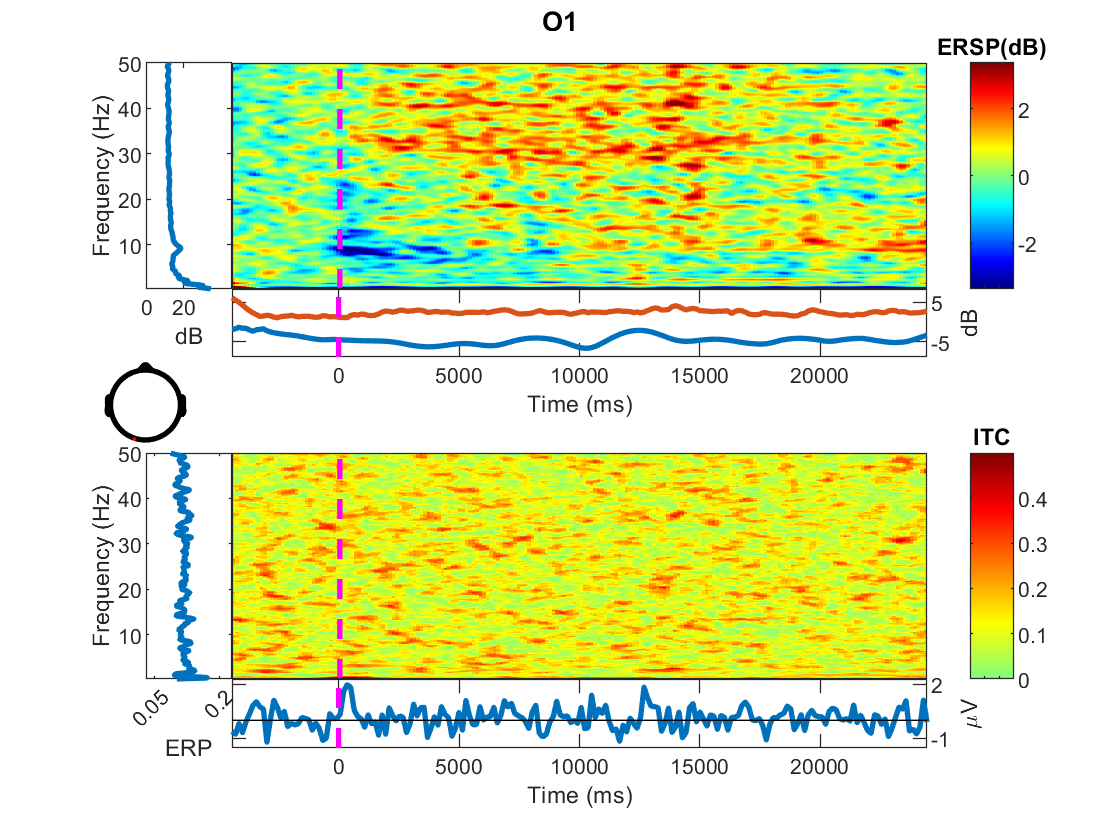

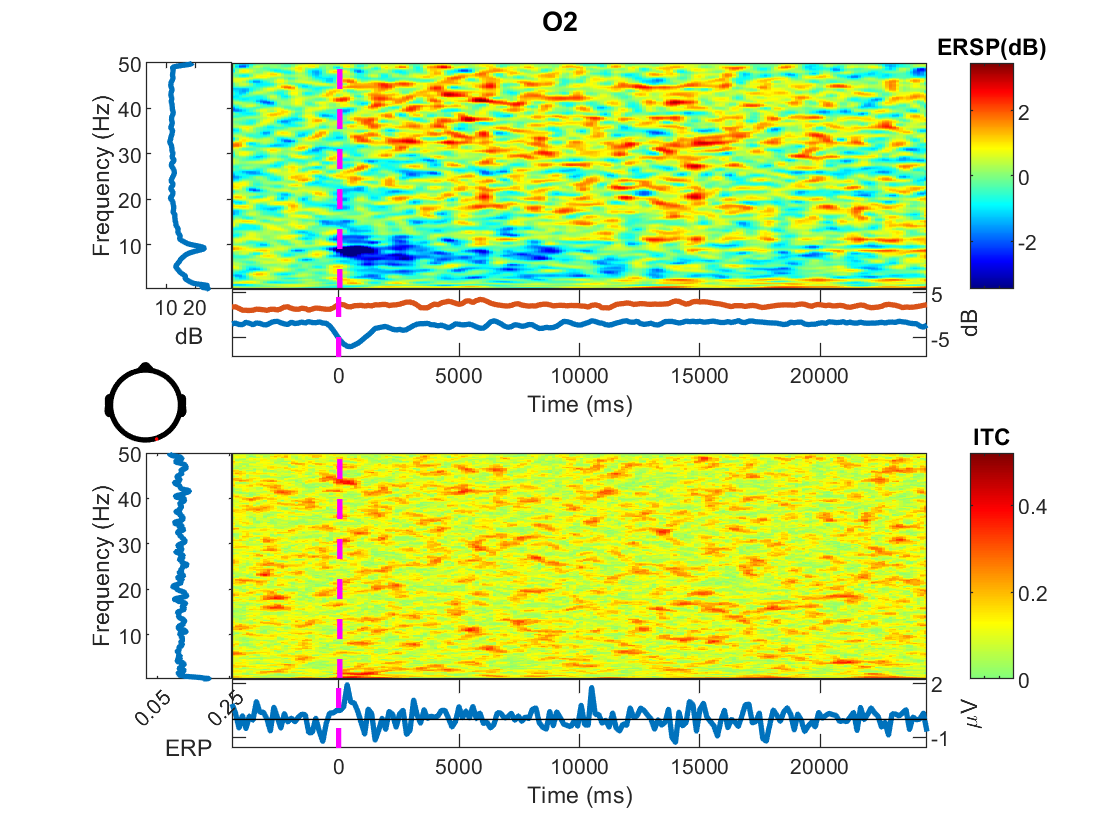
**Supplementary Fig. 1** Channel time frequency plots of initial recording for tinnitus onset.


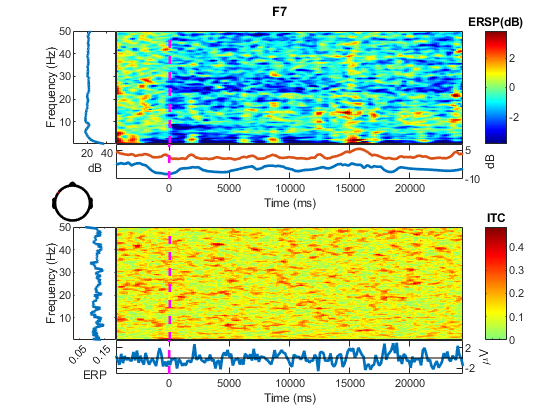

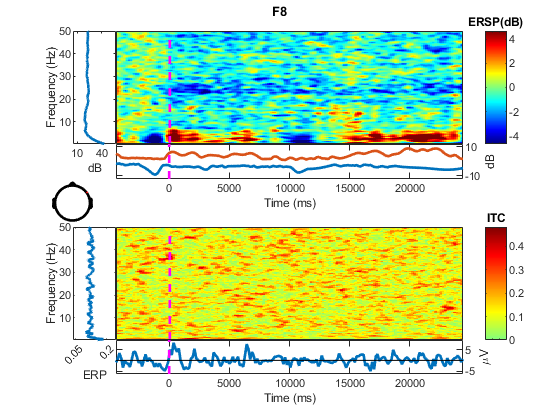

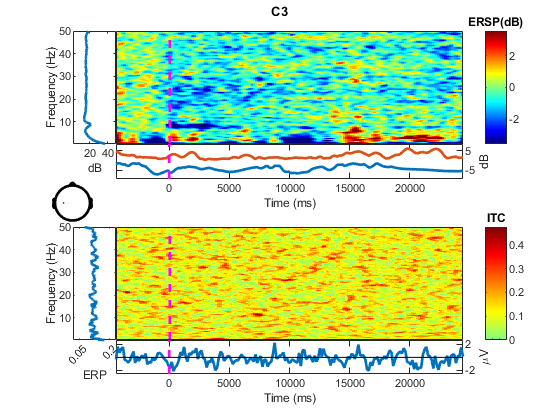

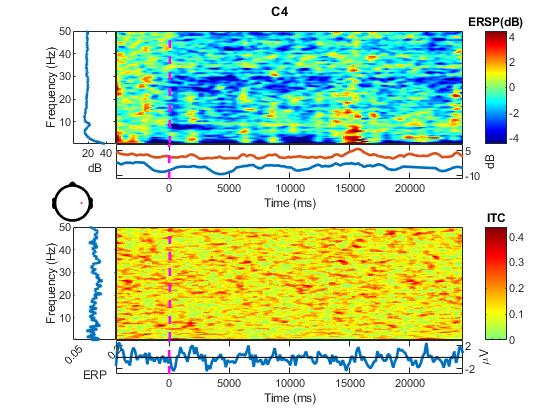

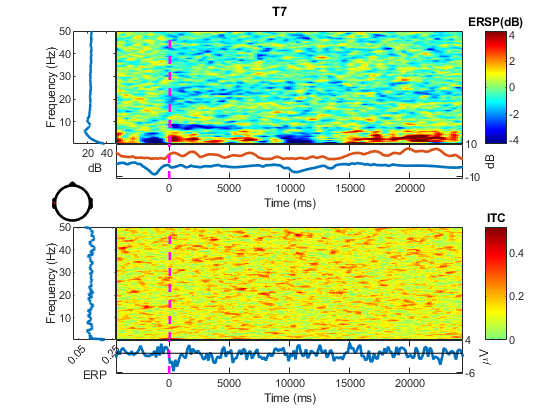

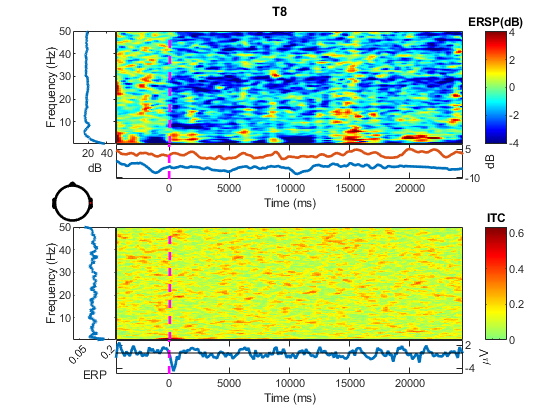

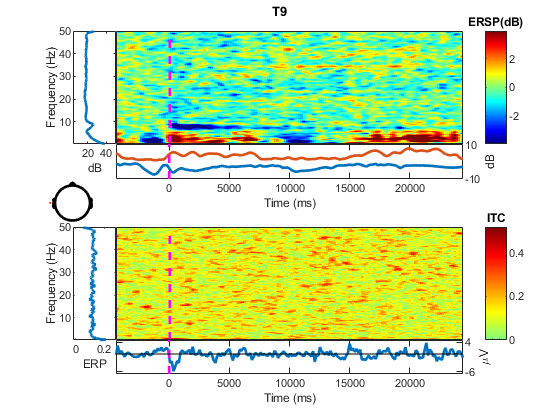

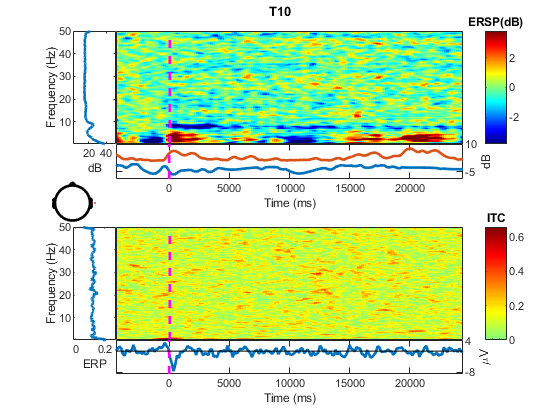

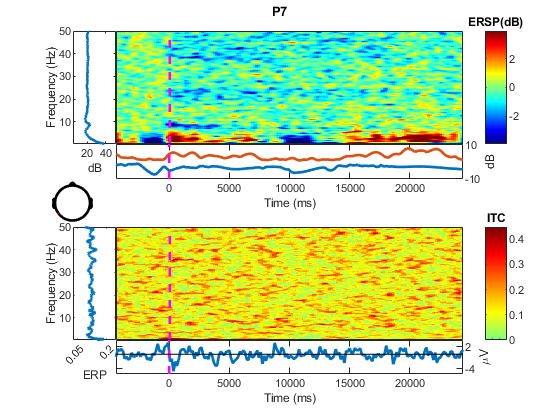

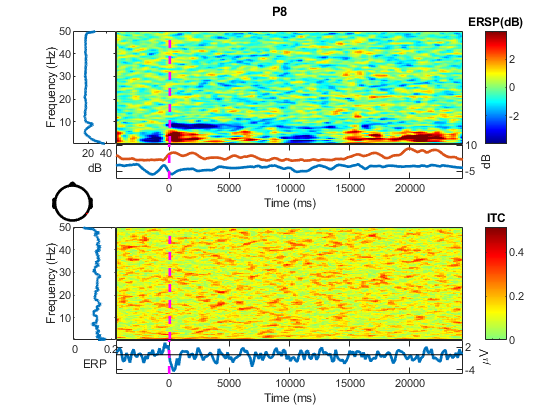

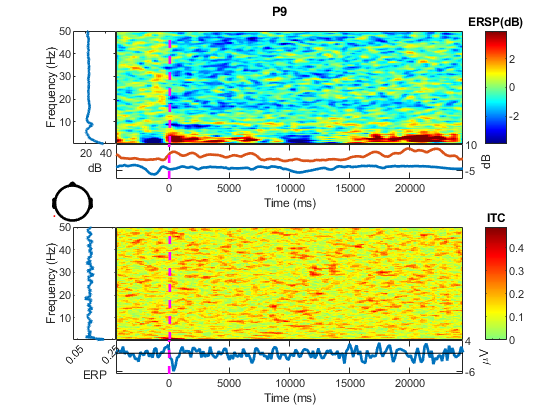

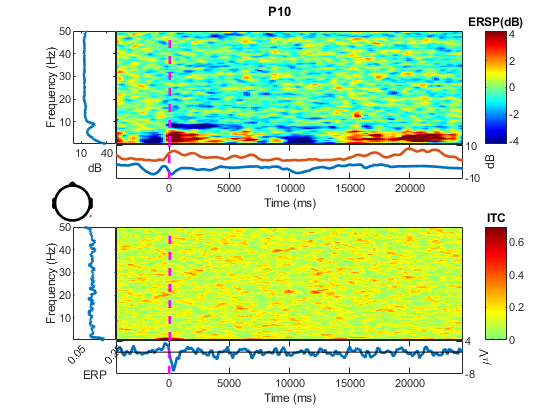

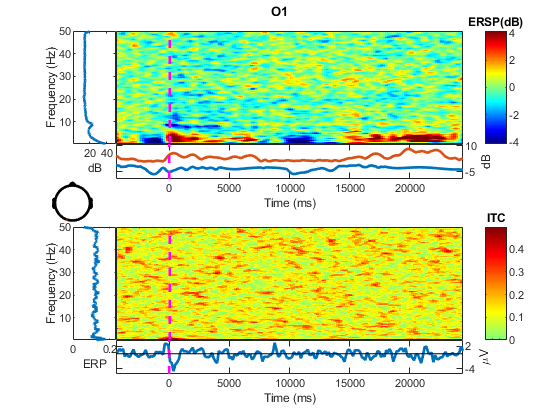

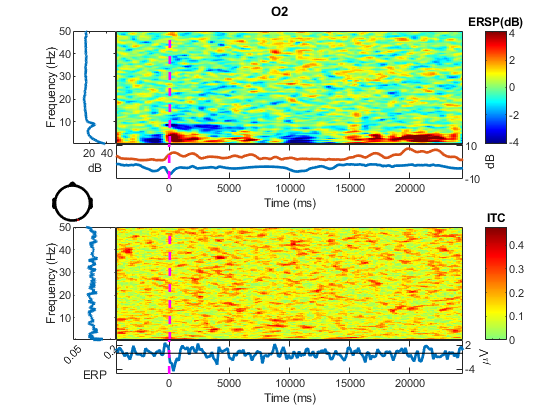


**Supplementary Fig. 2** Channel time frequency plots of second recording for tinnitus onset.


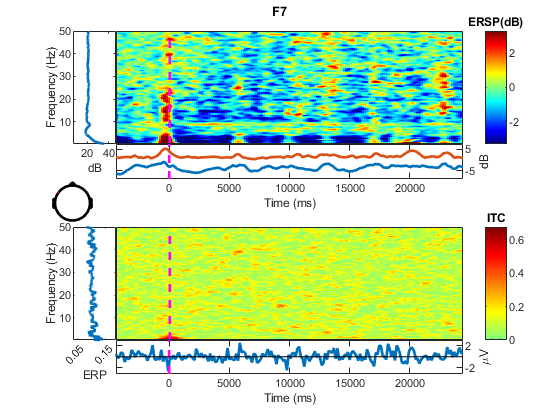

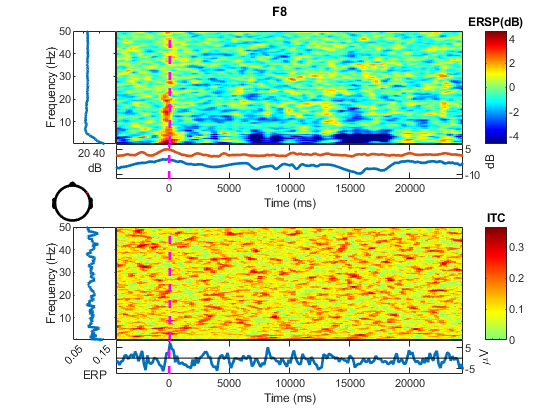

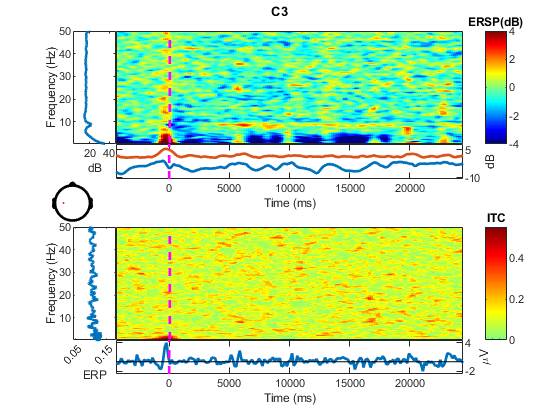

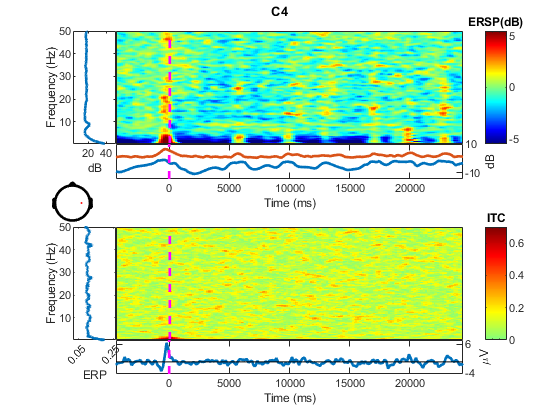

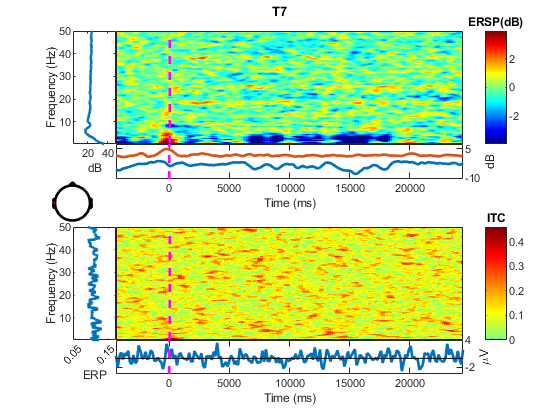

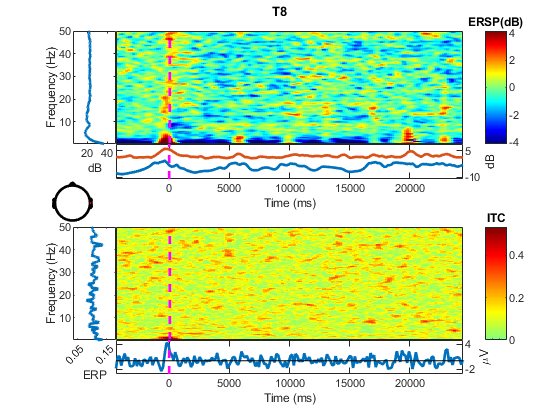

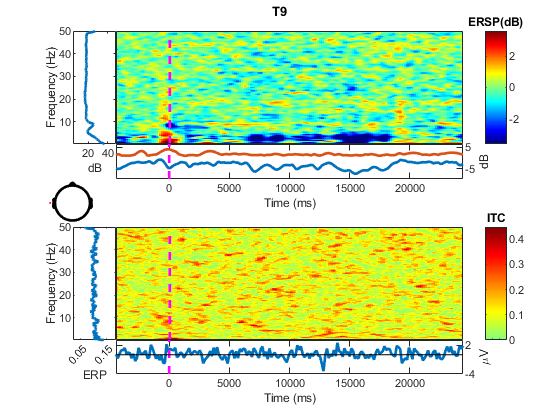

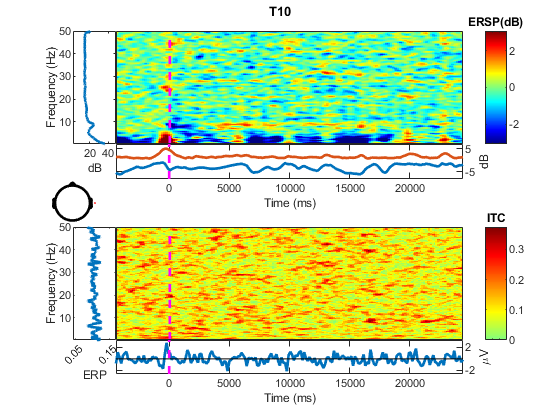

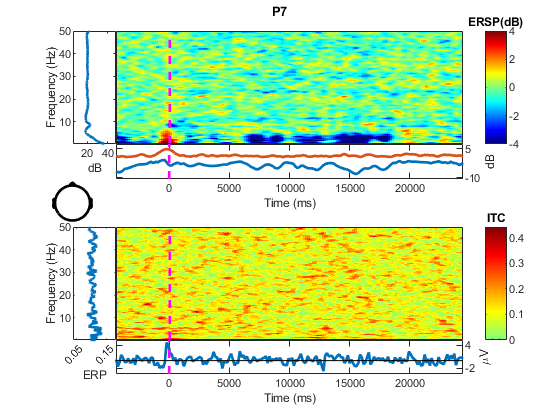

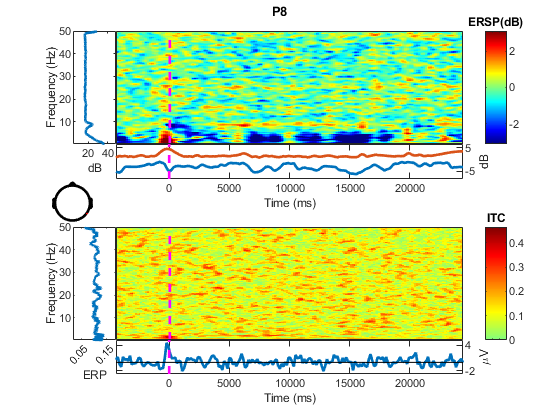

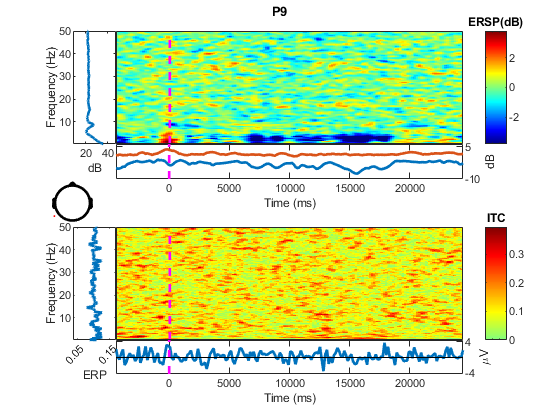

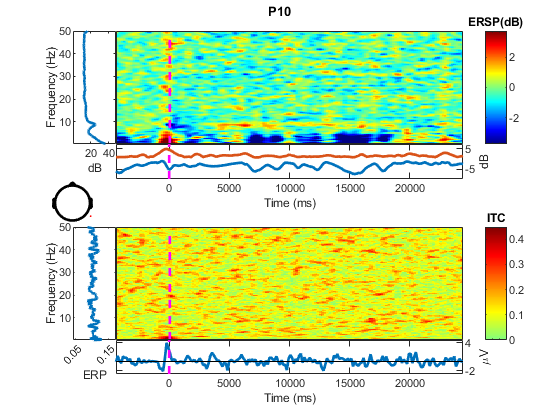

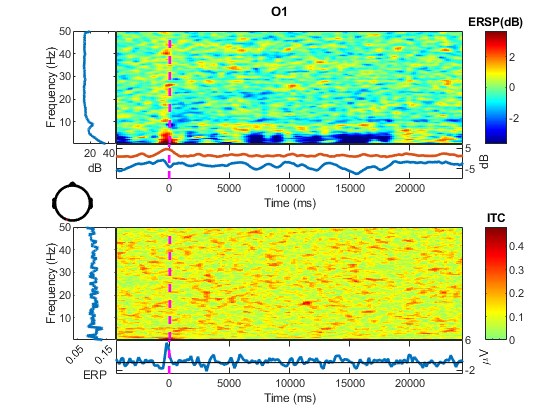

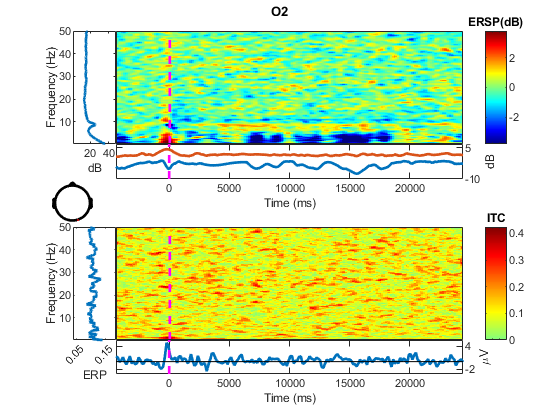


**Supplementary Fig. 3** Channel time frequency plots of second recording for finger extension.
